# Allele Age Under Non-Classical Assumptions is Clarified by an Exact Computational Markov Chain Approach

**DOI:** 10.1038/s41598-017-12239-0

**Published:** 2017-09-19

**Authors:** Bianca De Sanctis, Ivan Krukov, A. P. Jason de Koning

**Affiliations:** 1 University of Calgary, Cumming School of Medicine, Dept. of Biochemistry and Molecular Biology, Calgary, Alberta Canada; 2 University of Calgary, Cumming School of Medicine, Department of Biochemistry and Molecular Biology Graduate Program (Bioinformatics stream), Calgary, Alberta Canada; 3 University of Calgary, Cumming School of Medicine, Department of Medical Genetics, and Alberta Children’s Hospital Research Institute, Calgary, Alberta Canada

## Abstract

Determination of the age of an allele based on its population frequency is a well-studied problem in population genetics, for which a variety of approximations have been proposed. We present a new result that, surprisingly, allows the expectation and variance of allele age to be computed exactly (within machine precision) for any finite absorbing Markov chain model in a matter of seconds. This approach makes none of the classical assumptions (e.g., weak selection, reversibility, infinite sites), exploits modern sparse linear algebra techniques, integrates over all sample paths, and is rapidly computable for Wright-Fisher populations up to *N*_*e*_ = 100,000. With this approach, we study the joint effect of recurrent mutation, dominance, and selection, and demonstrate new examples of “selective strolls” where the classical symmetry of allele age with respect to selection is violated by weakly selected alleles that are older than neutral alleles at the same frequency. We also show evidence for a strong age imbalance, where rare deleterious alleles are expected to be substantially older than advantageous alleles observed at the same frequency when population-scaled mutation rates are large. These results highlight the under-appreciated utility of computational methods for the direct analysis of Markov chain models in population genetics.

## Introduction

Allele age is generally defined as the duration of time a mutant allele has been segregating in a population. The problem of calculating the expected age of an allele given its current population frequency is an important problem in population genomics (e.g., ref.^1^) with a long history of theoretical investigations (e.g., refs^2–6^; reviewed in ref.^7^). One reason that allele age remains an important problem is that the effects of selection and age can be highly confounded in terms of their influence on population frequency. That is, an allele may be at low frequency because it is deleterious or simply because it is young. Inferences about the fitness effects of segregating polymorphisms must therefore make some consideration of allele age, either explicitly or implicitly, and methods for inferring fitness impacts based on allele ages have even been proposed^8^.

The first theoretical analysis of allele age was developed by Kimura and Ohta^3^ using a continuous-time diffusion approximation to the age of a neutral allele in a finite population. Later work added consideration of selection^9^, yielding the well-known result that allele age is expected to be symmetric with respect to the direction of selection, and that neutral alleles are expected to be older than selected alleles observed at the same frequency (the “Maruyama effect” hereafter). Recently, an interesting exception to these classical results has been pointed out^10–12^. Mafessoni *et al*.^12^ showed that weakly selected rare alleles are expected to be about 5% older than neutral alleles observed at the same frequency, when heterozygote fitness is non-additive. This phenomenon appears to be an example of a more general behaviour recently termed ‘stochastic slowdown’^10^, where weak selection counter-intuitively prolongs, rather than shortens, the average time to absorption. It is important to understand the generality of these findings, since, as Mafessoni *et al*.^12^ point out, many new mutations arising in a population are expected to be recessive and weakly deleterious, and it is conceivable that this slowdown effect could thereby mislead attempts to make inferences about natural selection.

Previous investigations of allele age, and classical approaches in population genetics more generally, have required that mutation rates are assumed to be so slow that no additional mutations can occur during the segregation of an initial variant (implying that the population-scaled mutation rate, *θ*, is very small or ≈0). However, cases where this assumption is violated in nature are increasingly being reported, and it is likely in such cases that classical population genetic theory will be unreliable at best^13^. While in most eukaryotes, *θ* is estimated to be $\ll$0.05, several examples of so-called hyperdiverse eukaryotes are known with $\hat{\theta }$ between 0.05 and 0.15^14^. In bacteria, it is not uncommon for estimates of *θ* to be at the high end of this range or significantly larger. For example, Sung *et al*.^15^ reported average estimates taken from the literature of *θ* = 0.15 for *Helicobacter pylori* and 0.12 for *Salmonella enterica*, both of significant biomedical interest. Hughes *et al*.^16^ also reported $\hat{\theta }$ in *Pseudomonas syringae* to be 0.55. In addition, *θ* in some organisms including viruses and pathogens has been estimated to be much larger, by a variety of analytical methods, with estimates often exceeding 1. For example, *θ* in HIV-1 has been estimated to be between 10 and 369^17^ in one study, and >1 using the effective population size estimated by Pennings *et al*.^18,19^ together with mutation rates from other studies; similarly, *θ* in macaque monkeys infected with RT-SHIV (an engineered simian immundeficiency virus encoding human HIV-1 reverse transcriptase) has been estimated to be greater than one^20^. Other arguments that classical assumptions about *θ* may be violated in nature have also been recently put forward. For example, Messer and Petrov^21^ have highlighted that most known cases of molecular adaptation across diverse organisms show signatures of soft selective sweeps (but see ref.^22^), where adaptive alleles have multiple origins either by recurrent mutation or migration. These findings are potentially unexpected if evolution is strongly mutation-limited and may indicate that the effective population-scaled mutation rate is underestimated in many cases and/or that adaptation may tend to occur during periods of episodically large population size (and thus, high *θ*)^23^. We therefore decided to revisit the problem of calculating allele age based on population frequency under non-classical assumptions, and in particular to examine the impact of large values of *θ* on the expected age of an allele. For beneficial variants, the values of *θ* that we consider are expected to produce adaptive fixations that may have either multiple mutational origins or single origins^24^.

To study the effects of non-classical parameter ranges on allele age, we develop a new exact approach capable of rapidly computing moments of the allele age distribution under any absorbing discrete-time Markov chain model of population genetics. This approach exploits sparsity, parallelism, and modern computational architectures^25^, and is completely general with respect to the underlying model. It therefore requires none of the classical simplifying assumptions (e.g., weak selection, weak mutation, infinite sites, etc). For the purposes of the present study, we assumed a biallelic diploid Wright-Fisher model^26^ including bidirectional mutation, selection and dominance. Computationally, our solution mainly relies on back-substitutions using an LU decomposition of a sparse matrix derived from the model’s transition matrix, and does not use any matrix-matrix multiplications, which are computationally expensive. This computational implementation is similar to that in ref.^25^, where we applied sparse matrix techniques to the calculation of population genetic quantities such as the probability of fixation and sojourn times (but not allele age). To the best of our knowledge, this is the first computationally feasible, exact approach for computing allele age (or its moments) to be proposed. Calculation of the expected value and variance of allele age is fast, exact and scales easily to realistic population sizes (*N*_*e*_ ≈ 10^5^ for Wright-Fisher type models, and much larger for Moran models due to their greater sparsity; see Discussion). We have implemented this method in our software package Wright-Fisher Exact Solver, WFES^25^ (available at https://github.com/dekoning-lab/wfes/).

## Results

Using the approach outlined above and described fully in the Methods, we considered allele age and related quantities in a biallelic Wright-Fisher model including bidirectional mutation, selection, and dominance. For selection coefficient *s* and dominance coefficient *h*, the homozygous wildtype fitness was defined as 1, heterozygote fitness as 1 + *sh*, and homozygous mutant fitness as 1 + *s* (following standard definitions^26^). Bi-directional mutation was modelled in the Wright-Fisher transition matrix^26^, with extinction and fixation states assumed to be absorbing. This assumption implies a return process such that when a mutant frequency of 1 is attained, the population is returned to a frequency of 0 (equivalent to swapping the labels for the wild-type and mutant states); this allows properties of average trajectories to be easily calculated based on their starting or ending states.

In a biallelic diploid model, each individual may be either wild-type or mutant at each locus and chromosome. We define an “allele” here explicitly as the mutant genotype. Thus, allele age refers to how long the mutant state has been segregating in the population, starting from a population that was monomorphic for the wild-type state. By allowing mutation, we assume that an arbitrary number of new mutations could potentially arise in the population while an initial mutant is segregating, and thus the assumption of shared ancestry of all segregating mutants is not necessarily made. In the context of classical theory it may seem unnatural to consider allele age while including mutation. However, this is because classical theory makes the assumption that mutation cannot be recurrent, while there is no such prohibition in nature. Furthermore, even when *θ* is large, allele trajectories include long periods of time spent at the boundaries, and it therefore remains reasonable to demarcate the behaviour of such trajectories based on their visits to the boundaries. This may no longer be true when *θ* is so large that a population always contains all possible alleles ($\theta \gg 1$).

Except where otherwise specified, all results that follow are for a rare allele observed in *x* = 10 copies, sampled from an effective population size of *N*_*e*_ = 10,000 diploids. Forward and backward mutation rates were assumed equal. We consider a range of population-scaled mutation rates, *θ* = 4*N*_*e*_ *μ*, between *θ* = 0.0048 and *θ* = 0.96, where *μ* is the mutation rate per site per chromosome, and *N*_*e*_ the effective population size. Results obtained using values of *θ* that were two orders of magnitude smaller than *θ* = 0.0048 were largely similar (not shown).

### Validation by comparison to other methods

We first examined the correspondence between expected allele age determined by exact computation with the Wright-Fisher Markov model and the expected allele age approximated using Kimura and Ohta’s diffusion approach^3^. Since Kimura and Ohta’s method assumes no selection and no mutation, we ran our computations on a Wright-Fisher model having these same assumptions. Across a range of effective populations sizes and observed allele counts, the methods exhibited close correspondence (Table 1), where Kimura and Ohta’s method consistently overestimated allele age by a few generations.

**Table 1:** Expected neutral allele age determined by exact computation (this study) and by Kimura and Ohta’s^3^ diffusion approximation.

*N*_*e*_	*x*	Diffusion	Exact
1,000	10	106.5	103.73
5,000	10	138.29	134.99
10,000	10	152.09	148.56
20,000	10	165.92	162.16
50,000	10	184.23	180.16
1,000	100	630.68	628.65
5,000	100	930.34	927.8
10,000	100	1,064.99	1,062.22
20,000	100	1,201.30	1,198.30
50,000	100	1,382.93	1,379.63
1,000	1,000	2,772.59	2,771.02
5,000	1,000	5,116.86	5,115.03
10,000	1,000	6,306.80	6,304.77
20,000	1,000	7,566.93	7,564.69
50,000	1,000	9,303.37	9,300.85

No selection or mutation were assumed in the underlying Wright-Fisher model to ensure that the assumptions of both methods were compatible.

**Figure 1:**
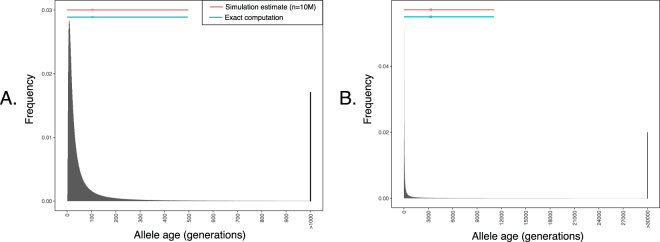
Representative neutral allele age probability distributions determined by simulation. Simulations for *N*_*e*_ = 1,000 were performed 10 million times with *s* = 0. As the allele age distributions have very long tails, the undisplayed portion of the tail is accumulated in the final bin. (**A**) *θ* = 0.01. (**B**) *θ* = 0.96. Intermediate values of *θ* are shown in Figs S2 and S3.

We next validated our method and its implementation by comparing results to allele age simulations that included selection, dominance, and mutation (Table 2). Allele age probability distributions can be approximated by simulation by reversing the direction of time in a Wright-Fisher model that is modified to have the same stationary distribution as the original (forward-time) transition matrix^27^. “Forward time” simulations of this reversed model can then be performed starting at the observed frequency, *x*/(2*N*_*e*_), and running until the beginning of the sample path (*p*/(2*N*_*e*_); see Methods for details). Simulations performed in this manner agreed well with the model-based computations across the entire parameter range. Allele-frequency probability distributions approximated by simulation are shown for a subset of cases in Fig. 1.

**Table 2:** Representative expected allele age and variance including selection, dominance and mutation determined by simulation and exact computation.

*θ*	2*N*_*e*_ *s*	*h*	Simulation		Exact	
			Mean	Std. Dev.	Mean	Std. Dev.
0.01	0	NA	106.67	391.64	106.39	389.57
0.05	0	NA	118.21	433.01	117.99	431.67
0.1	0	NA	135.17	493.47	134.91	491.67
0.5	0	NA	477.75	1,531.31	477.67	1,531.58
0.96	0	NA	3,315.99	7,775.71	3,320.94	7,791.84
0.01	−3	0.0	116.69	449.90	116.42	449.90
0.01	−3	0.5	97.04	319.34	96.86	317.74
0.01	−3	1.0	84.51	238.28	84.38	237.13
0.01	3	0.0	91.62	275.07	91.47	273.91
0.01	3	0.5	96.63	317.56	96.46	316.12
0.01	3	1.0	100.73	369.59	100.53	367.69
0.96	−3	0.0	4,746.56	9,021.43	4,742.61	9,011.70
0.96	−3	0.5	2,994.45	5,757.48	2,990.35	5,746.30
0.96	−3	1.0	1,996.64	3,813.41	1,994.75	3,808.97
0.96	3	0.0	729.07	2,090.27	728.74	2,088.35
0.96	3	0.5	773.22	2,759.72	773.03	2,758.71
0.96	3	1.0	933.51	4,004.62	932.89	4,004.79

A diploid population of *N*_*e*_ = 1,000 was assumed with *p* = 1 and *x* = 10.

### Computational advantages of the exact approach

Allele age simulations were implemented in C++ and parallelized, so that their runtimes would be reasonably fast (see https://github.com/dekoning-lab/allele_age_simulator/). Simulations were much more time consuming than the direct computation of the moments using our approach (e.g., 15 minutes versus 0.6 seconds for *θ* = 0.01; Table 3). As *θ* was increased, the simulations took increasingly more time both because the allele trajectories grew longer on average and because higher mutation rates also increased the variance in the duration of allele age trajectories. For *θ* = 0.96, running a 10 million replicate simulation over 32 cores took approximately 13 hours. On the other hand, the runtime for the exact matrix method was constant across different mutation rates and averaged about 0.5 seconds. Thus, when moments provide sufficient information, they can be obtained much more efficiently using our exact approach.

**Table 3:** Representative run-times (wall clock) for parallel computation of neutral allele age.

	Simulation*	Exact^†^
*θ*	Time (sec.)	Time (sec.)
0.01	911.12	0.58
0.05	1,021.56	0.61
0.1	1,190.46	0.60
0.5	5,143.14	0.44
0.96	46,723.40	0.64

*Simulations were run on 32 cores, parallelized across 10 M replicates. ^†^Exact calculation of the mean and variance of allele age was performed on the same machine using 8 threads. The number of threads in each case was chosen to approximately minimize wall clock time needed by each method.

### Direct demonstration of classical results

**Figure 2:**
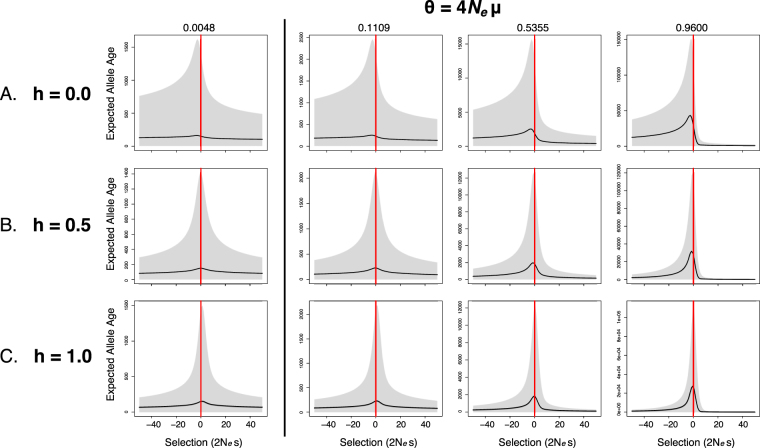
Expected allele age and variance as a function of selection, dominance, and mutation rate. All calculations were made for a rare allele (*x* = 10) assuming *N*_*e*_ = 10,000 diploids. When heterozygote fitness is non-additive, weakly selected alleles are expected to be older than neutral alleles observed at the same frequency ((**A**,**C**) left). When mutation is weak and heterozygote fitness is additive, allele age is symmetric with respect to the direction of selection ((**B**) left). When the mutation rate increases, an age imbalance with respect to the direction of selection appears (left to right). Full results over a larger grid of *θ* can be found in Fig. S1.

Several classical results pertaining to allele age can be directly obtained by examining expected allele age and variance as a function of selection (Fig. 2). It should be emphasized that these plots are neither probability distributions nor estimates. Rather, they are the exact moments of allele age derived directly from the Wright-Fisher model, as explained in the Methods section.

For rare alleles, the expected allele age has a large variance relative to the mean and the mean age is roughly symmetric with respect to the sign of the selection coefficient, with neutral alleles expected to be older than selected alleles (Fig. 2B, leftmost column; the Maruyama effect^9^). The symmetry of allele age with respect to the direction of selection is among the most conspicuous classical findings on allele age, and has been the subject of recent study, where different authors have both supported it using population genomic data^8^ and argued against it using simulations that included linkage^28^.

### Selective strolls and stochastic slowdowns

Recent work^10–12^ has convincingly demonstrated, using primarily simulation and diffusion theory methods, that weakly selected alleles are sometimes expected to be older than neutral alleles observed at the same frequency when fitness in heterozygotes is non-additive. This idea was termed “selective strolls” by Mafessoni *et al*.^12^, referring to the observation that selected variants may sometimes persist in a population slightly longer than neutral ones. Here we directly reproduce this effect for rare recessive alleles (*h* = 0), where it can be seen that weakly deleterious alleles are expected to be older than neutral alleles at the same frequency (Fig. 2A, leftmost column), and for dominant alleles (*h* = 1), where it can be seen that weakly advantageous alleles are expected to be older than neutral alleles at the same frequency (Fig. 2C, leftmost column). Consistent with the findings of Mafessoni *et al*.^12^, it is apparent that the selective stroll effect size is not very large and is on the order of about 5%.

### Recurrent mutation and age imbalance

Contrary to the Maruyama effect, for population-scaled mutation rates approaching *θ* ≈ 1 the mean allele age becomes strongly asymmetric around *s* = 0 (Fig. 2, c.f. left to right) such that weakly to moderately deleterious alleles can on average be substantially older than advantageous alleles at the same frequency. We refer to this previously unobserved phenomenon as “age imbalance”.

Under age imbalance, slightly deleterious alleles are also expected to generally be older than neutral alleles at the same frequency. This new example of stochastic slowdown is observed even when heterozygote fitness is additive (i.e., with *h* = 0.5). The effect size in this case is substantially larger than for the previously noted slowdowns with small *θ* (or *θ* = 0; ref.^12^). For example, expected extinction times for the oldest alleles with *h* = 0.5 are approximately 22.7% longer than for neutral alleles.

**Figure 3:**
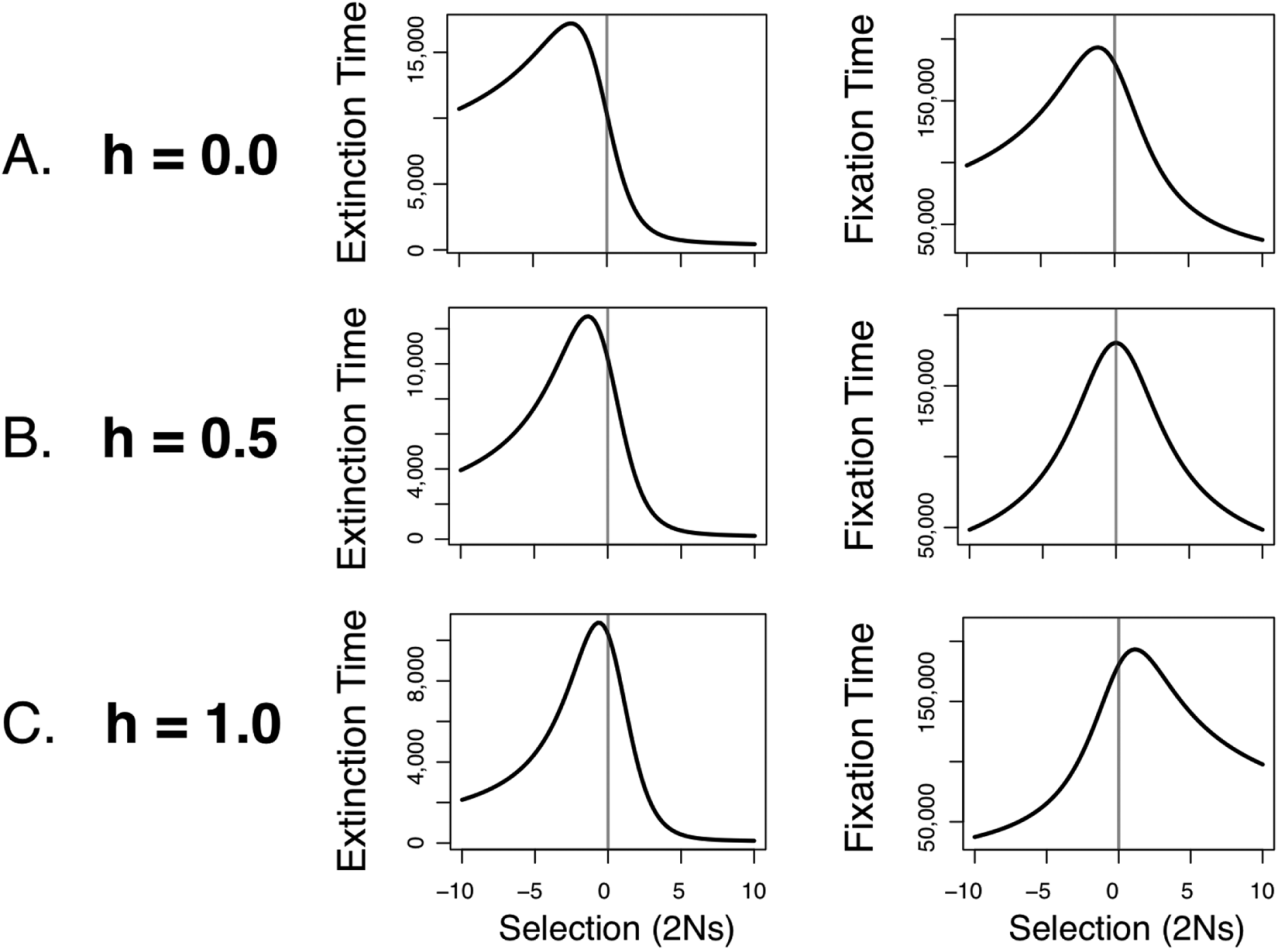
Expected extinction and fixation times when mutation is strong (*θ* = 0.96) calculated by exact computation with the Wright-Fisher Markov model^25^. Parameter values used were the same as in Fig. 2. Note the strong asymmetry with respect to the direction of selection, contrary to the classical result of Maruyama and Kimura^32^.

Rare recessive alleles (*h* = 0) under recurrent mutation and large *θ* (Fig. 2, right) experience the same effect but to an even greater degree. Recessivity and fast mutation appear to have a similar and mutually reinforcing effect on both age imbalance and the stochastic slowdown under weak selection. Both selective stroll and age imbalance results appear to be explained primarily by the average time to extinction (Fig. 3, left), which indicates that when mutation rates are bidirectionally fast, weakly deleterious alleles counter-intuitively take longer to go extinct than do advantageous (or neutral) alleles. For *h* = 0 extinction times are even longer for deleterious recessive alleles than for those with *h* = 0.5, but now the expected fixation times also show a similar imbalance with respect to the direction of selection (Fig. 3, c.f. A and B), which accentuates the stochastic slowdown further. Remarkably, the expected time to extinction for the oldest, weakly selected recessive alleles is about 66.9% longer than for neutral alleles (Fig. 2A, left). The same results for *h* = 1 are shown in Fig. 3C, where fixation times are shifted to the right rather than the left, which seems to largely cancel out the stochastic slowdown caused by the left-shifted extinction times.

**Figure 4:**
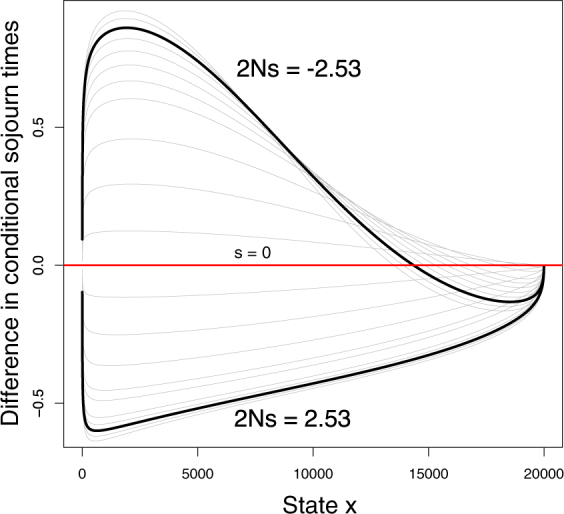
Difference in conditional sojourn times (compared to neutral) for selected alleles going to extinction. Curves span approximately 2*N*_*e*_ *s* = {−3, 3}; *h* = 0, *θ* = 0.96. Top: increasing selection against the mutant allele up to the critical point 2*N*_*e*_ *s* = −2.53 counter-intuitively increases sojourn times by prolonging residency in low frequency classes. Bottom: increasing selection favouring the mutant allele decreases the length of extinction sojourns. Bold: 2*N*_*e*_ *s* = ±2.53. The maximum of the extinction time curve in Fig. 3A is at −2.53.

To help explain Fig. 3, we also calculated the conditional sojourn times for mutants that go to extinction, and compared these to sojourn times for neutral variants (Fig. 4). For deleterious alleles, we see that the time spent at low frequencies increases as we move away from 2*N*_*e*_ *s* = 0 until 2*N*_*e*_ *s* = −2.53 is reached; the stronger selection is within this parameter range, the more extinction sojourns are dominated by residency at lower frequencies compared to neutral. While this trend is expected since negative selection opposes increases in allele frequency, it is surprising that the net change in non-neutral sojourn times is positive. That is, the increased time at low frequencies surpasses the decreased time spent at high frequencies, resulting in longer sojourns overall. This phenomenon has also been reported for previously noted stochastic slowdowns^11^.

### Allowing the starting number of copies to vary

When population-scaled mutation rates are very high it can become plausible that an originating mutation enters the population in several copies (i.e., that it simultaneously occurs in several individuals). For example, when *θ* = 0.96, the average number of mutations entering the population per generation is 0.96/2 = 0.48, so on average there will be a new mutation every two generations. The probability of a population generating multiple copies of the mutant allele in a single generation, assuming mutations are Poisson distributed, is ≈0.38. This may pose problems for any method for calculating allele age, since when the likelihood of a population simultaneously generating more than one mutant becomes non-negligible, the starting number of copies, *p*, should be integrated out.

To integrate over *p* we consider the probability of starting in *p* copies, given that $p\sim Poisson\,(\lambda =\theta \mathrm{/2)}$. This can be easily implemented in our computational procedure starting at Equation 8 by reusing the LU decomposition of (*I* − *Q*)^*T*^, which does not depend on *p* (see Methods). Since this decomposition is by far the most computationally expensive operation, the integrated solution is trivially harder than when assuming a single starting copy. In addition, since the probability of large numbers of mutations occurring in the same generation will typically be negligible, we define a threshold *ε* such that only starting configurations with a probability greater than *ε* are considered. Below, we assumed *ε* = 10^−5^.

**Figure 5:**
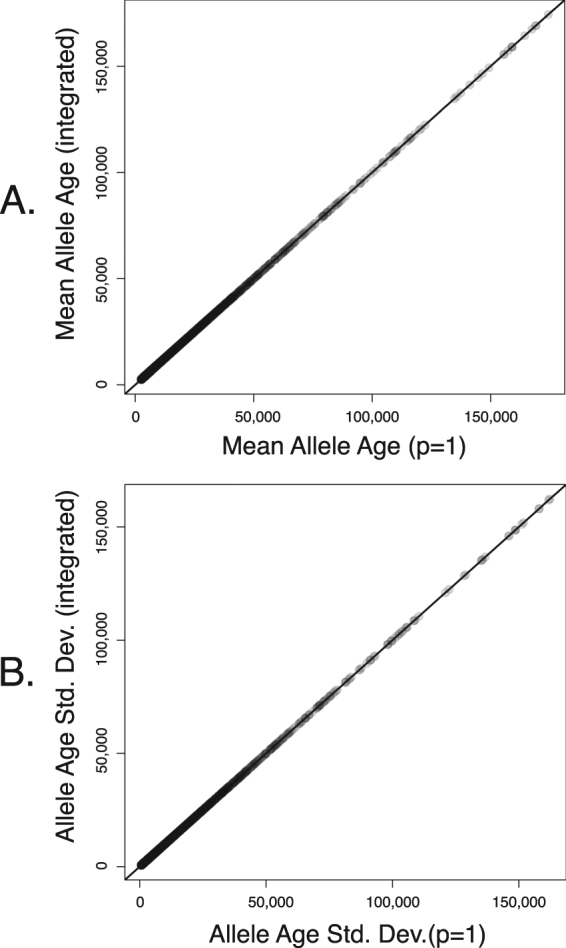
Effect of integrating out uncertainty in *p*. The integral (summation) was taken to a finite number of terms such that all values with *p* ≥ 10^−5^ were considered. Points represent all parameter combinations considered in Fig. 2.

In Fig. 5 we show the effect of numerically integrating over *p* when *θ* = 0.96 for the range of mutation rates, selection coefficients, and dominance coefficients considered throughout the manuscript. In most cases, the results were identical at better than three to four decimal places, and only began to diverge slightly when *θ* was very large (i.e., *θ* = 0.96). It is possible that other statistics of the Markov process might change more than this as a function of *p*, and thus to be conservative one may choose to always integrate over *p* (particularly since this adds only seconds to the compute time). However, we conclude that assuming *p* = 1 (as is done by convention in all previous studies of allele age that we are aware of) is likely to introduce no bias unless *θ* is quite large (i.e., $\gg$1).

## Discussion

Computational population genetics approaches offer the relatively straightforward ability to explore parameter ranges or assumptions that may be inaccessible to classical theory. Usually simulations are used to address scenarios where the assumptions of classical theory may be violated. However, simulations can often be slow, require long runtimes to obtain precise estimates for rare events, and can scale poorly to large populations. An alternative computational approach is to find a class of models whose properties can be interrogated directly, without the need for simulation. For example, Steinruecken *et al*.^29^ recently showed how the transition density function of biallelic Wright-Fisher diffusions^30^ could be approximately computed, eliminating the need for a variety of simulations (although allele age has not been considered in this framework). Here we have shown that even the exact computational analysis of biallelic Markov models (including Wright-Fisher models) can be made efficient enough to often eliminate the need for either simulations or diffusion approximations in the first place. Markov chain models are typically discounted early in the lifecycle of a population genetic investigation in favour of diffusion approximations, since they are widely viewed as impractical to work with due to their large and potentially unwieldy state spaces. Contrariwise, here and elsewhere^25^, we have shown that judicious computation, sparsity, and parallelism can be together exploited to rather surprising effect, making exact computation under general Markov models not only tractable but capable of generating new insights with ease. Working directly with the underlying Markov models of population genetics has a number of advantages. For example, when strong mutation is included, absorbing boundaries can artificially become inaccessible in a diffusion. There is no corresponding problem when studying the unapproximated Markov chain. In addition, diffusion approaches cannot easily describe behaviours at the absorbing boundaries (but see ref.^31^).

One of the most appealing aspects of this computational population genetics approach is that it is general with respect to underlying modelling assumptions, as long as they can be expressed as a finite absorbing Markov chain. This approach also has several advantages over simulations, including fast runtimes that are relatively insensitive to modelling assumptions (Table 3), and exact results (within machine precision) even for small effects or rare events that would otherwise require long-run, high replicate simulations to study. For a population size of *N*_*e*_ = 10,000, exact calculation of the expected allele age and variance, absorption probabilities and times, and conditional sojourn times, takes only about 6.5 seconds using 16 Intel E5-2670 cores (2.60 GHz) in our reference implementation^25^. Models with greater sparsity are even faster and can scale much better. For example, the same analysis under a comparable Moran model takes only about 0.25 seconds^25^.

The method proposed for calculating allele age is based on the efficient computation of the moments of the probability distribution of allele ages. It is therefore appropriate to view these quantities not as estimates, but as exact results for a given model. An advantage of this approach is that the expected value of the allele age probability distribution will more often be much closer to the true allele age than would a maximum likelihood estimator, since the age distributions are both highly skewed and very long tailed (see Fig. 1). A potential disadvantage is that we must assume that the true population frequency is known without error. In cases where it is not, error in the observed frequency could be accounted for by computing allele age for a range of population frequencies centred on the observed value.

As shown in Fig. 2, classical allele age results^3,9,32^ can be easily obtained for general population genetic models with our approach. We also reproduced exact representations of recently discovered effects, such as “selective strolls”, which have a smaller effect on expected allele age when mutation rates are low (also see ref.^12^). By exploiting the generality of our approach, we discovered new evidence for a stochastic slowdown that occurs when bidirectional mutation is fast, such that rare, weakly deleterious alleles are expected to be substantially older than neutral alleles. In the most extreme case, average extinction times for the oldest alleles were 22% and 68% longer than for neutral alleles (for *h* = 0.5 and *h* = 0, respectively). Finally, we found that when relaxing the assumption of weak mutation, a large age imbalance arises with respect to selection, such that rare deleterious alleles are expected to be old and rare advantageous alleles very young. This may be explained in part by the expectation that with strong mutation pressure and positive selection, allele frequencies will rise rapidly following origination. When this is true, the best explanation for a beneficial allele being rare is that it only arose quite recently. This expected rapid rise in mutant frequency under strong mutation and positive selection may also be responsible for the much faster extinction times for beneficial alleles compared to deleterious ones (Fig. 3: left), since the longer beneficial alleles persist, the more likely their frequencies are to be pushed upwards towards fixation. Consequently, the mutants that go to extinction are most likely to do so quickly.

A potential limitation of our approach to calculating allele age is that we have assumed equilibrium demography with constant population size. However, this is a limitation of our implementation rather than of the method itself. One solution to this problem is to consider instantaneous switches among different population sizes under a Markov-modulated model. By virtue of our sparse linear algebra approach, this would only be linearly more difficult than the constant population size approach. It could also have advantages over existing diffusion theory methods^33^, for example, by faithfully modelling an increase in the population mutation rate during population growth that includes the effect of recurrent mutation. Such considerations may be important for understanding adaptation in organisms with “boom and bust” population dynamics^23^. We leave exploration of these ideas for future work.

## Methods

### Theory

Let *X*(*t*) be an absorbing discrete-time Markov chain with known transition matrix *P* and state-space defined by the number of copies of a mutant allele in a population of *N*_*e*_ effective diploid individuals. Let *Q* be the submatrix of *P* that contains only transient-to-transient state transitions. Assume that the current number of mutant alleles *x* is a transient state, so the allele in question is neither extinct nor fixed. We also assume that the allele entered the population at a specific frequency *p*/(2*N*_*e*_), where *p* is a transient state (we later show how this assumption can be relaxed). In practice, we consider *p* = 1 unless stated otherwise.

The probability of transitioning from state *p* to state *x* in time *t* is simply ${P}_{p,x}^{t}$, or equivalently ${Q}_{p,x}^{t}$ since both *p* and *x* are transient states. Since the Markov chain is absorbing,

1 $$\sum _{t=0}^{\infty }\,{Q}_{p,x}^{t}={(I-Q)}_{p,x}^{-1}$$

is finite^34^, where *I* is the identity matrix. This finiteness allows us to fix *x* and *p* and specify a probability distribution of the allele age.

2 $${f}_{p,x}(t)=\frac{{Q}_{p,x}^{t}}{{\sum }_{t=0}^{\infty }{Q}_{p,x}^{t}}=\frac{{Q}_{p,x}^{t}}{{(I-Q)}_{p,x}^{-1}}$$

A complete measure theoretic construction of this distribution can be found in the supplementary material S1 Appendix. The exact moments of this distribution can be written in terms of the matrix *Q* by using matrix sum identities. We show the first three below using [*A*]_*b*,*c*_ to denote the entry in the *b*-th row and *c*-th column of matrix *A*.

3 $${\mu }_{1}=\sum _{t=0}^{\infty }\,t\,{f}_{p,x}(t)=\frac{{\sum }_{t\mathrm{=0}}^{\infty }t{Q}_{p,x}^{t}}{{\sum }_{t=0}^{\infty }{Q}_{p,x}^{t}}=\frac{{[Q{(I-Q)}^{-2}]}_{p,x}}{{[(I-Q{)}^{-1}]}_{p,x}}$$

4 $${\mu }_{2}=\sum _{t=0}^{\infty }\,{t}^{2}{f}_{p,x}(t)=\frac{{\sum }_{t=0}^{\infty }{t}^{2}{Q}_{p,x}^{t}}{{\sum }_{t=0}^{\infty }{Q}_{p,x}^{t}}=\frac{{[Q(I+Q)(I-Q{)}^{-3}]}_{p,x}}{{[(I-Q{)}^{-1}]}_{p,x}}$$

5 $${\mu }_{3}=\sum _{t\mathrm{=0}}^{\infty }\,{t}^{3}{f}_{p,x}(t)=\frac{{\sum }_{t=0}^{\infty }{t}^{3}{Q}_{p,x}^{t}}{{\sum }_{t=0}^{\infty }{Q}_{p,x}^{t}}=\frac{{[Q({Q}^{2}+4Q+\mathrm{1)(}I-Q{)}^{-4}]}_{p,x}}{{[(I-Q{)}^{-1}]}_{p,x}}$$

The expected allele age is given by *μ*_1_, and the variance is given by ${\mu }_{2}-{\mu }_{1}^{2}$.

It is interesting, and relevant if the reader wishes to compute higher moments than those listed above, to notice that the *k*-th moment *μ*_*k*_ is closely linked to the matrix polylogarithm function *Li*_−*k*_(*Q*) by the following equation.

6 $${\mu }_{k}=\frac{{[L{i}_{-k}(Q)]}_{p,x}}{{[(I-Q{)}^{-1}]}_{p,x}}$$

where

7 $$L{i}_{-s}(z)=\sum _{k\mathrm{=1}}^{\infty }\,{z}^{k}{k}^{s}={(z\frac{\partial }{\partial z})}^{s}(z{\mathrm{(1}-z)}^{-1})$$

Combining equations 6 and 7 therefore allows for the rapid symbolic computation of the closed-form expressions for any moment *μ*_*k*_.

### Implementation

Computation of the moments in Equations 3, 4 and 5 can be greatly simplified. This simplification requires obtaining a single LU decomposition of a sparse matrix and using it to solve multiple linear systems by back-substitution. This computational approach is similar to our approach in ref.^25^, where it was applied to the calculation of quantities such as the probability of fixation and sojourn times.

The first step is to calculate the LU decomposition of (*I* − *Q*)^*T*^, where *T* denotes transpose. LU decomposition has a theoretical time complexity on the same order as matrix multiplication, and thus can be as large as *O*(*n* ^3^) floating point operations for a dense *n* × *n* matrix. However, much faster solutions are possible for sparse matrices, which scale in terms of the number of non-zero entries (e.g., refs^35,^ ^36^). For Wright-Fisher models, *Q* and hence (*I* − *Q*)^*T*^, are typically very sparse (at machine precision), and thus a potentially large time savings can be obtained by exploiting this sparsity. Computation of the LU decomposition is by far the most time-intensive step, but we find it is still feasible for population sizes around 10^5^ on typical workstation computers as of the time of writing^25^. As noted earlier, much larger effective population sizes can be easily considered with the more sparse Moran model.

The second step is to use forward and back substitution to solve multiple linear systems. Given the LU decomposition, this is quite fast and typically requires only a few seconds. First we solve for *M*_1_ in

8 $${(I-Q)}^{T}{M}_{1}={e}_{p}$$

where *e*_*p*_ is the *p*-th column of the identity matrix. Note that ${M}_{1}^{T}$ is the *p*-th row of (*I* − *Q*)^−1^, so that the *x*-th entry of *M*_1_ is in fact ${(I-Q)}_{p,x}^{-1}$ as required in the denominator of Equations 3 and 4.

Next, we use the same LU decomposition to solve for *M*_2_ in

9 $${(I-Q)}^{T}{M}_{2}={M}_{1}$$

Notice that

10 $${((I-Q{)}^{2})}^{T}{M}_{2}={(I-Q)}^{T}{(I-Q)}^{T}{M}_{2}={(I-Q)}^{T}{M}_{1}={e}_{p}$$

so that ${M}_{2}^{T}$ is actually the *p*-th row of (*I* − *Q*)^−2^. We next take the dot product of ${M}_{2}^{T}$ with the *x*-th column of *Q*, which we call *Q*_*x*_.

11 $${M}_{2}^{T}\cdot {Q}_{x}={[(I-Q{)}^{-2}Q]}_{p,x}={[Q{(I-Q)}^{-2}]}_{p,x}$$

which is what was required in the numerator of Equation 3.

We repeat the procedure and solve for *M*_3_ in

12 $${(I-Q)}^{T}{M}_{3}={M}_{2}$$

Again, we have

13 $${((I-Q{)}^{3})}^{T}{M}_{3}={(I-Q)}^{T}{(I-Q)}^{T}{M}_{2}={(I-Q)}^{T}{M}_{1}={e}_{p}$$

so that ${M}_{3}^{T}$ is the *p*-th row of (*I* − *Q*)^−3^. In order to compute the numerator of the second moment, we also need the *x*-th column of *Q*(*I* + *Q*), which we call *A*_*x*_. Note this does not in any way necessitate a full matrix multiplication, as we require only the *x*-th column. Although this is potentially an expensive *O*(*n* ^2^) computation, in practice, sparsity makes it trivially easy. Now we have

14 $${M}_{3}^{T}\cdot {A}_{x}={[(I-Q{)}^{-3}Q(I+Q)]}_{p,x}={[Q(I+Q)(I-Q{)}^{-3}]}_{p,x}$$

as required in the numerator of Equation 4.

Hence we have calculated all necessary components of the expected value and variance as given in Equations 3 and 4.

The computation of higher moments can be easily implemented as well. To do this, one would first use equations 6 and 7 to obtain closed-form expressions for the needed moments. We recommend using a factored form of the expression so that matrix multiplication is never required in the implementation (it is a convenient property of the polylogarithm that all closed-form expressions of *Li*_−*s*_(*z*) factor completely over the reals). The implementation would then require extending the above algorithm as needed, i.e. iteratively solving

15 $${(I-Q)}^{T}{M}_{k+1}={M}_{k}$$

for *M*_*k*+1_, where ${M}_{k}^{T}$ is the *p*-th row of (*I* − *Q*)^−*k*^.

We have implemented this approach for the first two moments in our software package Wright-Fisher Exact Solver, WFES^25^ (available at https://github.com/dekoning-lab/wfes/). In practice it takes only seconds to minutes to calculate the relevant quantities for population sizes under *N*_*e*_ = 100,000.

As an aside, we note that the full probability distribution can also be feasibly approximated for small *N*_*e*_ to an arbitrary degree of precision by taking the summation in equation 2 to some large finite value.

## Simulations

In order to simulate a distribution of allele ages, we must reverse the process, i.e. use the reversed absorbing Markov chain. Specifically, the simulation will start at state *x* and essentially run backwards in time until it hits state *p*. It will then either keep going, or stop with a probability equal to the probability that the current visit to state *p* is the beginning of the chain (when the mutation first entered the population). This backwards simulation can be done by creating a reversed transition matrix and running it in a forwards simulation.

We use the method presented in Chae *et al*.^27^, which is as follows. The states of the reversed absorbing Markov chain are {1, 2, …, 2*N*_*e*_ − 2, 2*N*_*e*_ − 1, stop}, where the stop state is absorbing and all others are transient. The reversed Markov chain does not regard fixation or extinction as absorbing states, and in fact does not allow transition to these states at all.

Let *P*′ be the matrix of transition probabilities of the reversed absorbing Markov chain. In its canonical form,

16 $$P=(\begin{array}{cc}Q^{\prime}  & R^{\prime} \\ 0 & I\end{array})$$

We have

17 $${Q}_{j,k}^{^{\prime} }=\frac{{Q}_{k,j}{N}_{p,k}}{{N}_{p,j}}\,{\rm{and}}\,{R}_{j,i}^{^{\prime} }=(\begin{array}{ll}{N}_{p,p}^{-1} & {\rm{if}}\,j=p,\,i={\rm{stop}}\\ 0 & {\rm{otherwise}}\end{array}$$

where *Q* and *N* are the transient-to-transient state transition matrix and the fundamental matrix, respectively, of the original Markov chain. (Note that *N* here is used by convention to represent the fundamental matrix and has no relationship to *N*_*e*_ defined above).

## Electronic supplementary material

Supplementary information and figures
