# Supplementary material for: Allele Age Under Non-Classical Assumptions is Clarified by an Exact Computational Markov Chain Approach
Source: Sci Rep. 2017 Sep 19;7:11869. doi: 10.1038/s41598-017-12239-0 (PMC5605573; doi:10.1038/s41598-017-12239-0)

Supplementary Information for: "Allele Age Under Non-Classical Assumptions is Clarified by an Exact Computational Markov Chain Approach"

Bianca De Sanctis, Ivan Krukov, Jason de Koning

S1 Appendix. The purpose of this appendix is to present a measure theoretic construction of the distribution of allele age, for a fixed p and x, where p and x are transient states of the Markov Chain. This is necessary to prove the correctness of the method presented.

Let the sample space Ω be the set containing all finite realizations of the Markov Chain which begin with p and end with x. We write an arbitrary element of Ω as the ordered tuple

$$\omega = (p, a_1, a_2, ..., a_n, x) \in \Omega \tag{1}$$

where $n \in \{0, 1, 2, ...\}$ is arbitrary and finite and a_i are transient states in the Markov Chain for $1 \leq i \leq n$. It is worth noting that if $p = x$, then $(p) \in \Omega$ as well. The set Ω is (infinitely) countable because for each fixed length n, there are only finitely many realizations of the Markov Chain. Define $F = 2^{\Omega}$, the set of all subsets of Ω, so that F is trivially a σ-algebra satisfying the necessary conditions (that is, $\Omega \in F$ and F is closed under complement and countable union).

To define a probability measure $P : F \to [0, 1]$, we first define P on each singleton. Define $P(\{(p)\}) = 1/c$ if $p = x$, and for all other $\omega \in \Omega$,

$$P(\{\omega\}) = P((p, a_1, a_2, ..., a_n, x)) = \frac{1}{c} Q_{p,a_1} Q_{a_1,a_2} ... Q_{a_n,x} \tag{2}$$

where c is a constant that will allow $P(\Omega) = 1$. Since every element of F other than the empty set is a disjoint union of singletons, extending P beyond singleton sets to the rest of F is then just a matter of applying countable additivity and allowing $P(\emptyset) = 0$.

Let us find the constant c. Define a function $l : \Omega \to \mathbb{R}$ such that $l(\omega)$ gives the number of transitions that occurred in that specific realization, for example $l((p, x)) = 1$. Let $\Omega_m \subset \Omega$ be the subset containing all elements with m transitions, that is,

$$\Omega_m = \{\omega \in \Omega \mid l(\omega) = m\} \tag{3}$$

For a fixed m, we have

$$P(\Omega_m) = \sum_{\omega \in \Omega_m} P(\{\omega\}) = \frac{1}{c} Q^m_{p,x} \tag{4}$$

by the Chapman-Kolmogorov equation, where the entry is taken after the matrix power.

Therefore,

$$1 = P(\Omega) = P\left(\bigcup_{m=0}^{\infty} \Omega_m\right) = \sum_{m=0}^{\infty} P(\Omega_m) = \frac{1}{c} \sum_{m=0}^{\infty} Q^m_{p,x} \tag{5}$$

$$= \frac{1}{c} (I - Q)^{-1}_{p,x} \tag{6}$$

where I is the identity matrix, so that $c = (I - Q)^{-1}_{p,x}$. Thus we have fully defined the probability space. It is now easy to define the random variable $Y : \Omega \to \mathbb{R}$ as $Y(\omega) = l(\omega)$, with probability mass function

$$f_Y(y) = \begin{cases} Q^y_{p,x}/(I-Q)^{-1}_{p,x} & \text{if } y \in \{0,1,2,3,...\} \\ 0 & \text{otherwise} \end{cases} \tag{7}$$

Supplementary Figure S1

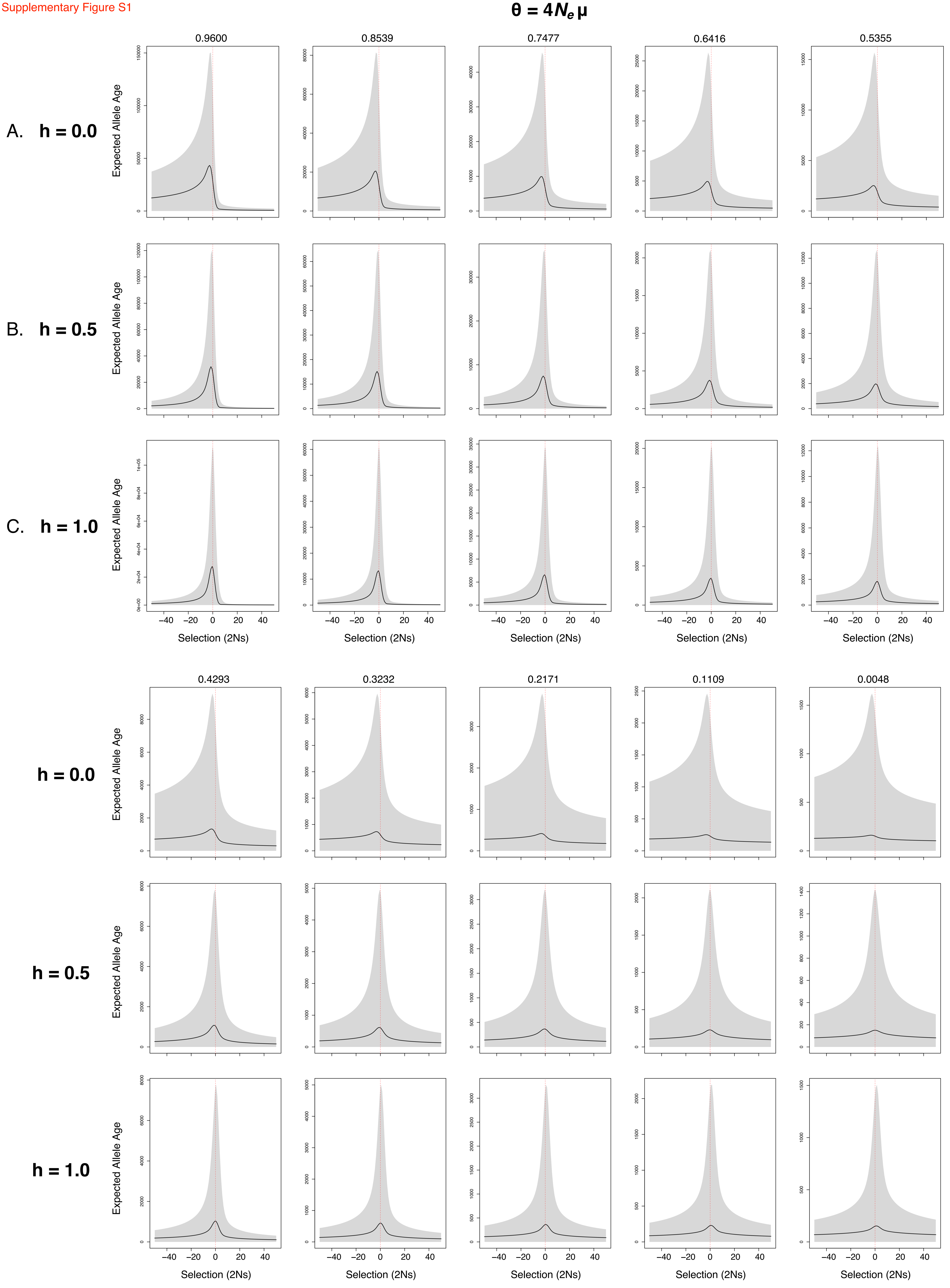

Supplementary Figure S2

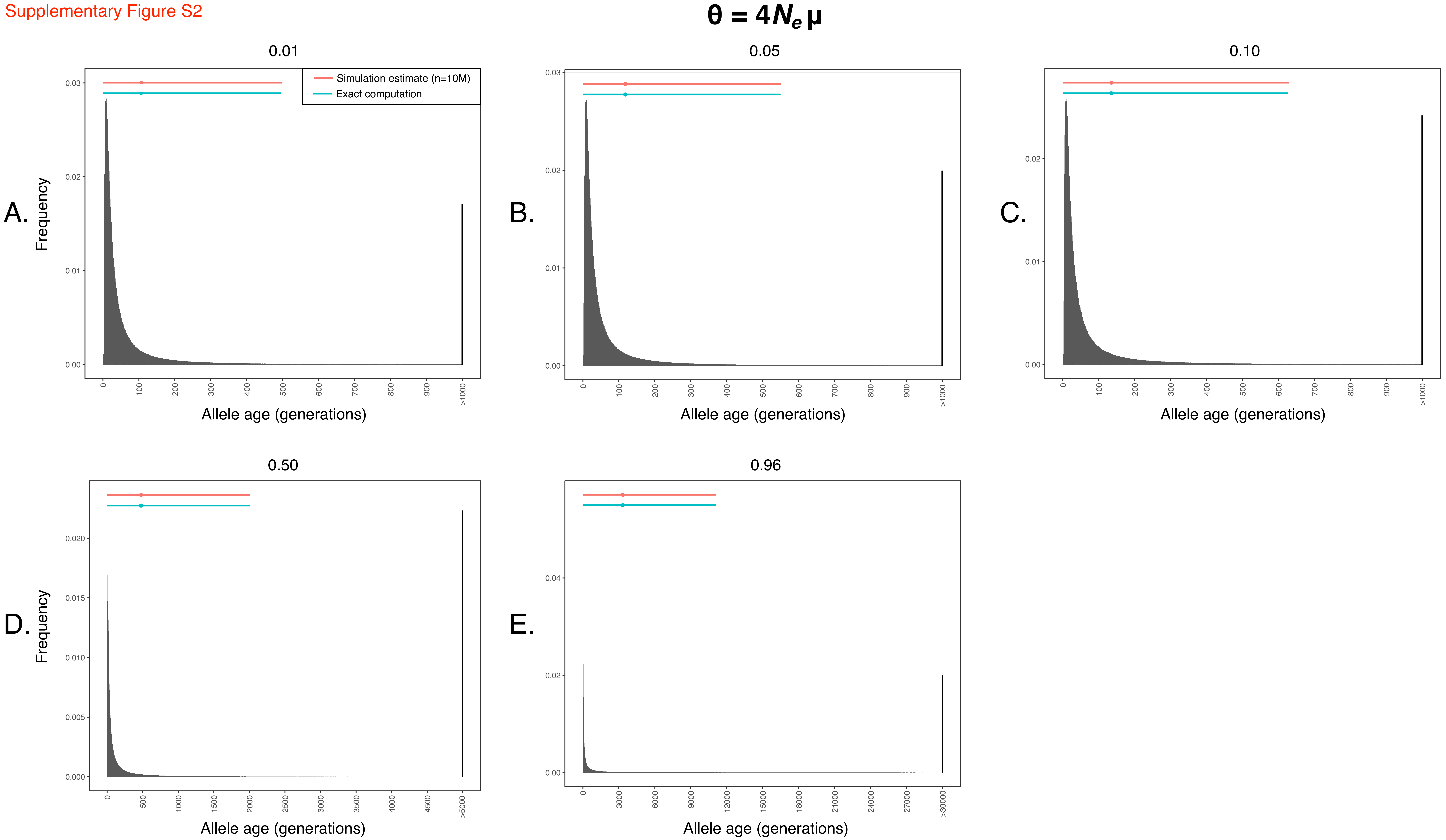

Supplementary Figure S3

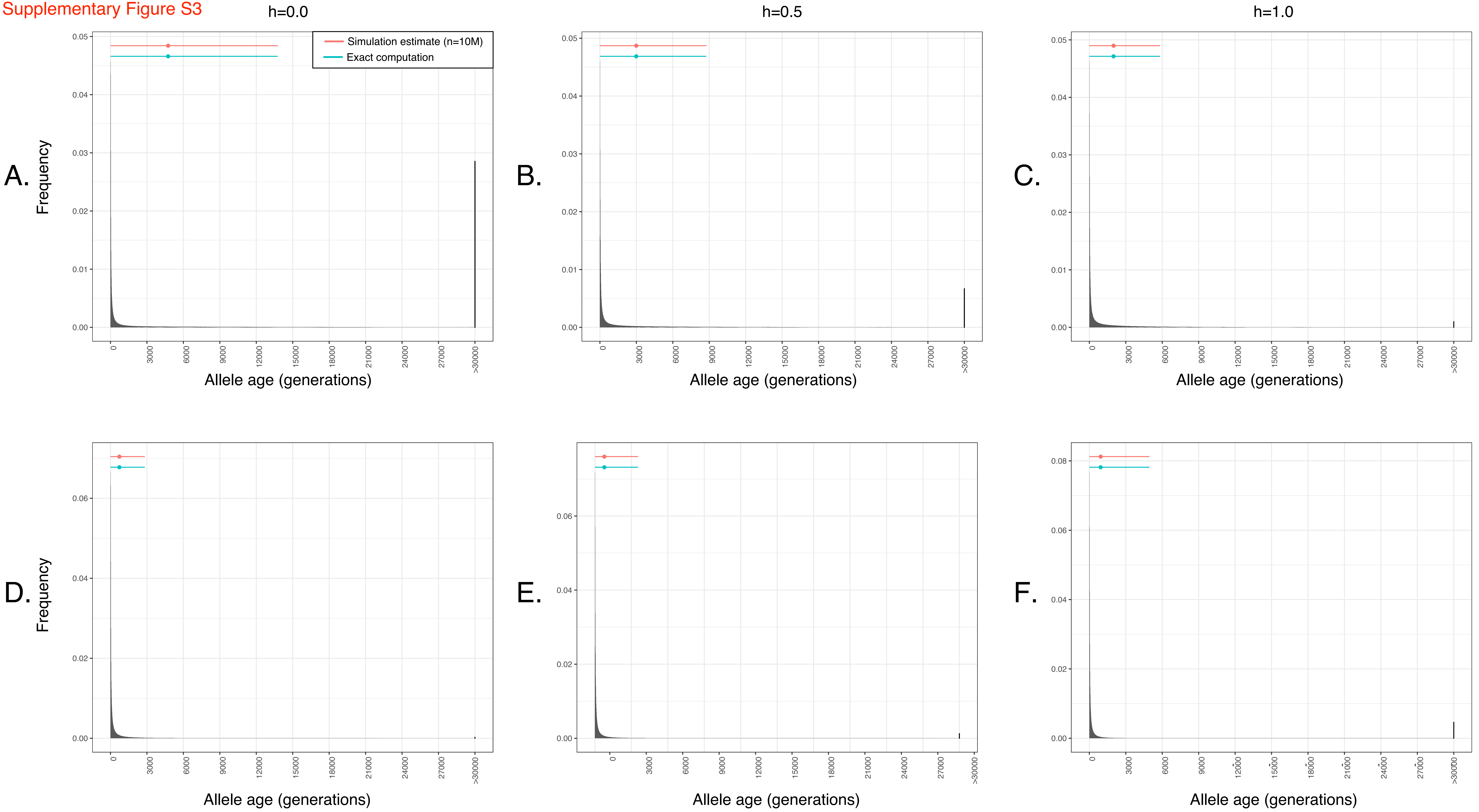

Supplementary Figure S4

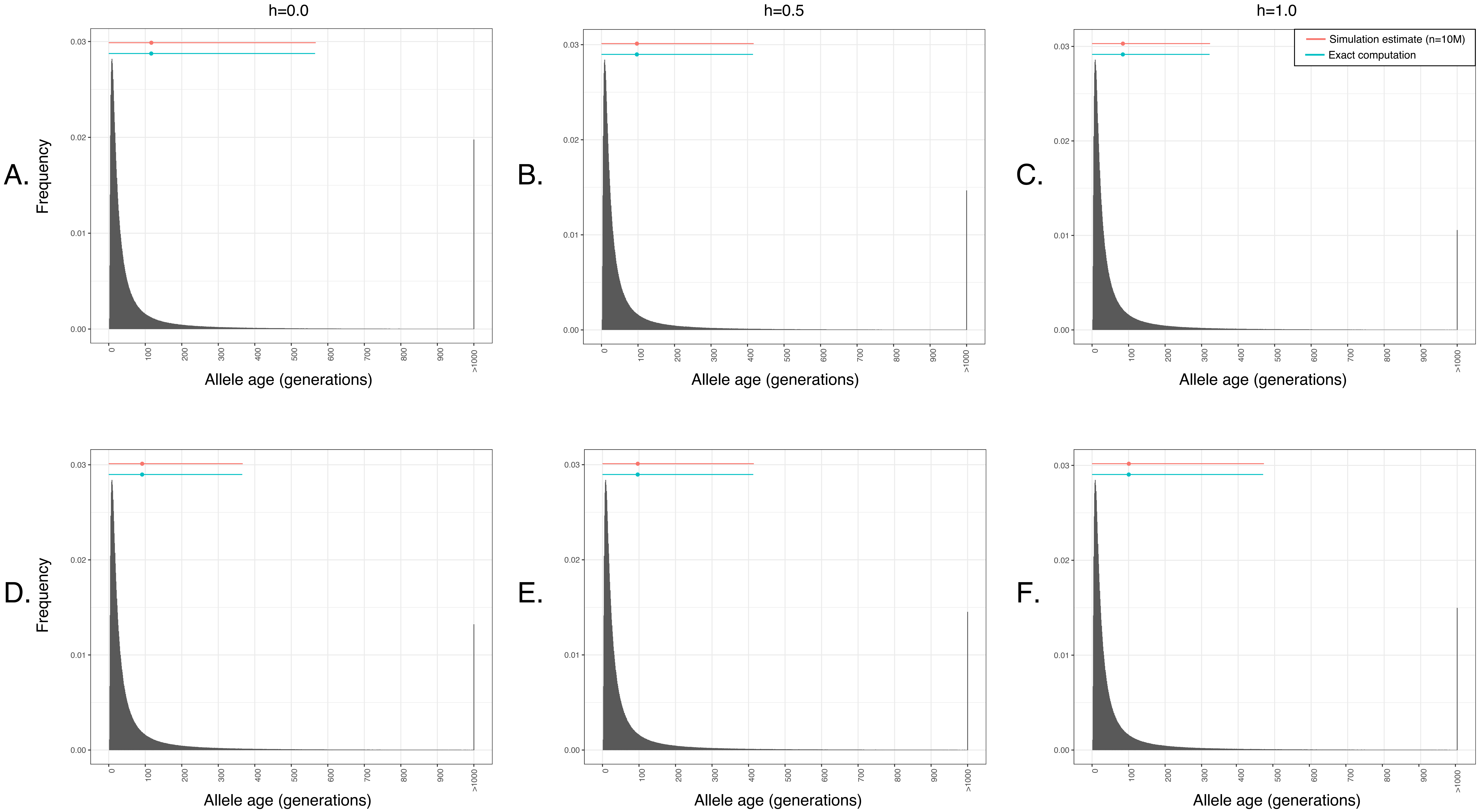

Supplement: Supplementary file 1 — Supplementary information and figures [file 41598_2017_12239_MOESM1_ESM.pdf]
